# Curcumin derivative WZ35 inhibits tumor cell growth via ROS-YAP-JNK signaling pathway in breast cancer

**DOI:** 10.1186/s13046-019-1424-4

**Published:** 2019-11-08

**Authors:** Lihua Wang, Canwei Wang, Zheying Tao, Liqian Zhao, Zheng Zhu, Wencan Wu, Ye He, Hong Chen, Bin Zheng, Xiangjie Huang, Yun Yu, Linjun Yang, Guang Liang, Ri Cui, Tongke Chen

**Affiliations:** 1 School of Ophthalmology and Optometry, Eye Hospital, Wenzhou Medical University, Wenzhou, 325000 Zhejiang China; 2 State Key Laboratory of Ophthalmology, Optometry and Visual Science, Wenzhou, 325000 Zhejiang China; 3 Affiliated Yueqing Hospital and School of Pharmaceutical Sciences, Wenzhou Medical University, Wenzhou, 325035 Zhejiang China; 4 School of Medicine, Shanghai Jiaotong University, Shanghai, 200030 China; 5 Laboratory Animal Centre, Wenzhou Medical University, Wenzhou, Zhejiang China; 6 School of Pharmaceutical Sciences, Wenzhou Medical University, Wenzhou, 325035 Zhejiang China

**Keywords:** Breast Cancer, WZ35, YAP, ROS, JNK, Mitochondrial dysfunction

## Abstract

**Background:**

Breast cancer is the most prevalent cancer among women worldwide. WZ35, an analog of curcumin, has been demonstrated to remarkably improve the pharmacokinetic profiles in vivo compared with curcumin. WZ35 exhibits promising antitumor activity in gastric cancer, HCC, colon cancer. However, antitumor effects of WZ35 in breast cancer and its underlying molecular mechanisms remain unclear.

**Methods:**

CCK8, Flow cytometry and transwell assays were used to measure cell proliferation, cell cycle arrest, apoptosis, cell migration and invasion. We constructed xenograft mouse model and lung metastasis model to assess the antitumor activities of WZ35 in vivo. To explore the underlying molecular mechanisms of WZ35, we performed a series of overexpression and knockdown experiments. The cellular oxygen consumption rates (OCRs) was measured to assess mitochondrial dysfunction.

**Results:**

We found that treatment of breast cancer cells with WZ35 exerts stronger anti-tumor activities than curcumin both in vitro and in vivo. Mechanistically, our research showed that WZ35 induced reactive oxygen species (ROS) generation and subsequent YAP mediated JNK activation in breast cancer cells. Abrogation of ROS production markedly attenuated WZ35 induced anti-tumor activities as well as YAP and JNK activation. In addition, ROS mediated YAP and JNK activation induced mitochondrial dysfunction in breast cancer cells.

**Conclusion:**

Our study showed that novel anti-cancer mechanisms of WZ35 in breast cancer cells and ROS-YAP-JNK pathway might be a potential therapeutic target for the treatment of breast cancer patients.

## Background

Breast cancer is a heterogeneous disease that is considered as the most frequently diagnosed cancer among women with high mortality and morbidity worldwide. The incidence of breast cancer is increasing year by year in developed countries and developing countries [1, 2]. Hormone-responsive breast cancer could benefit from currently available endocrine therapy, however, breast cancer cells lacking hormone receptors generally using chemotherapeutic drugs, such as taxol and doxorubicin [3]. These chemotherapeutic drugs exhibit high dose-limiting toxicity to tumor cells as well as normal cells, which limit their clinical usage [4]. In addition, endocrine therapy resistance has become the biggest limitation for treatment of breast cancer [3]. Thus, searching for less toxic and effective therapeutics is urgently needed.

Curcumin is a natural polyphenolic compound, obtained and purified from the powdered rhizome of the *Curcuma longa* L [5]. Substantial studies have reported that curcumin plays an essential role in anti-bacterial, anti-proliferative, anti-inflammatory, antioxidant, anti-carcinogenic and anti-amyloidogenic effects in vitro and in vivo through targeting various molecules [6, 7]. Meanwhile, it has been reported that anti-cancer activity of curcumin is mainly through the stimulation of the innate and adaptive immune systems [8–10]. However, poor bioavailability in vivo of curcumin per se has impeded its use in cancer therapy [11, 12]. To solve this problem, a new compound of curcumin analog WZ35, 1-(4-hydroxy-3-methoxyphenyl)-5-(2-nitrophenyl) penta-1,4-dien − 3-one, has been designed and synthesized by our lab. WZ35 has been proved possessing anti-cancer activities in gastric cancer by activating ROS-dependent ER stress and JNK mitochondrial pathways [13]. Similar anti-cancer effects have been found in colon cancer and hepatocellular carcinoma (HCC) [14, 15]. However, the function of WZ35 in breast cancer remains unclear. There is considerable evidence showing that loss of Hippo pathway or overexpression of YAP/TAZ was associated with human cancers including lung, liver and intestine cancers through promoting cancer cell growth and suppressing cell apoptosis [16–19]. On the contrary, hyperactivation of YAP is associated with a better prognosis in breast cancer patients, which suggests that YAP might act as a tumor suppressor in breast cancer [20].

Here, we demonstrated that WZ35 inhibits breast cancer cell growth, migration and invasion through activating ROS-YAP-JNK pathway. We further found that ROS-YAP-JNK pathway was involved in mitochondrial dysfunction in breast cancer cells. Our results suggest that WZ35 might be an effective therapeutic agent and targeting ROS-YAP-JNK pathway could be a potential therapeutic method for the treatment of breast cancer patients.

## Materials and methods

### Reagents and antibodies

Curcumin was purchased from Sigma (St. Louis, MO). WZ35, an analogue of curcumin, was synthesized by our lab and its structure has been described previously [13]. Oligomycin, carbonylcyanide-p-trifluorometh oxyphenylhydrazone (FCCP), antimycin A and rotenone were purchased from sigma (St. Louis, MO). CCK-8 (CK04) were obtained from DOJINDO. Horseradish peroxidase (HRP)-conjugated anti-rabbit (BL003A) and anti-mouse (BL001A) immunoglobulin glucose were purchased from Biosharp (Anhui, China). DCFH-DA ROS detection kit (S0033), NAC and SP600125 (S1876) were obtained from Beyotime (Haimen, China). BCA protein assay kit (23227) and Pierce ECL western blotting substrate (34095) were obtained from Thermo Scientific (Waltham, MA). Primary antibodies POLG (ab128899), EF4 (GUF1, ab171161), β-actin (ab8226) were obtained from abcam (HKSP, New Territories, HK). Phospho-SAPK/JNK Thr183/Tyr185 (#4668), JNK (#9252), E-cadherin (#8834S), N-cadherin (#13116S), cleaved Caspase-3 (#9664S), LATS1 (#3477), MOB1 (#13730), p-MOB1 (#8699), MST1 (#3682), MST2 (#3952), SAV1 (#13301), Nrf1 (#69432), Nrf2 (#12721), YAP (#4912), Bcl-2 (#2870), p-Akt^ser473^ (#4060), Cyclin B1 (#4138), Akt (#9272) and GAPDH (#5174) were obtained from Cell Signaling Technology (USA). MMP-2 (sc13594) and MMP-9 (sc21736) were purchased from Santa Cruz Biotechnology, Inc. P21 (10355–1-AP) were obtained from Precision Technologies Group (Chicago, USA).

### Clinical specimens

Twenty two primary breast cancer specimens and their adjacent tissue counterparts were obtained from the First Affiliated Hospital of Wenzhou Medical University and informed consents were obtained from the patients. All studies and procedures involving human tissues were in accordance with the ethical standards of the institutional and/or national research committee and with the 1964 Helsinki Declaration and its later amendments or comparable ethical standards.

### Cell culture and transfection

Human breast cancer cell lines, MDA-MB-231 and Hs578T cells were purchased from the Institute of Biochemistry and Cell Biology, Chinese Academy of Sciences. BEAS-2B cells were obtained from American Type Culture Collection (CRL-9609). Cells were cultured with dulbecco’s modified eagle medium (DMEM) (Life Technologies) supplemented with 10% fetal bovine serum (FBS) (Life Technologies) and antibiotics (100 U/mL penicillin and 100 μg/mL streptomycin) at 37 °C in a humidified incubator with 5% CO_2_. The MCF10A cells were purchased from the ATCC (CRL-10317). Cells were grown in DMEM/F12 supplemented with 5% horse serum, 0.5 mg/ml hydrocortisone, 20 ng/ml EGF, 10 μg/ml insulin, 100 ng/ml cholera toxinand and cultured at 37 °C with 5% CO2. The si-YAP and YAP overexpressing vector were transfected into MDA-MB-231 cells with lipofectamine 2000.

### Cell proliferation assay

Cell proliferation was evaluated by the CCK8 assay. MDA-MB-231, Hs578T, BEAS-2B and MCF10A cells were initially plated in 96-well plates at 5 × 10^3^ cells per well, cultured overnight. MDA-MB-231 and Hs578T cells were treated with curcumin or WZ35 with concentrations of 5, 10 and 20 μg/mL. For cytotoxicity assay, BEAS-2B and MCF10A cells were treated with WZ35 with concentrations of 0.5, 1, 2.5, 5, 7.5, 10 μg/mL and 0.25, 0.5, 1, 2.5, 5, 7.5 and 10 μg/mL respectively. MDA-MB-231 cells were used as control group. After 24 h or 48 h, 10 μl CCK8 reagent was added into each well and incubated for 3 h, followed by measurement of the optical density (OD) at a wavelength of 450 nm using a microplate reader (Bio-Rad). Curcumin, WZ35 and SP600125 were dissolved in 0.03% DMSO; NAC was dissolved in PBS and diluted with a complete medium containing 10% FBS to the final concentration. After treating with drug for 24 h, 48 h or 72 h, Cell proliferation was evaluated by the CCK8 assay.

### Flow cytometry analysis for cell cycle, apoptosis and ROS determination

MDA-MB-231 cells were plated in 6-well plates at 1 × 10^5^ cells per well. After treatment with curcumin (10 μg/mL) and WZ35 (10 μg/mL) for 24 h, cells were harvested with trypsin and washed with PBS, then resuspended with 70% prechilled ethanol, and stored at − 20 °C overnight. After 24 h, the fixed cells were collected and stained with propidium iodide using Cycle Test Plus DNA Reagent Kit (Becton Dickinson, San Jose, CA). The stained cells were then analyzed for DNA content using FACS caliber (BD, Franklin Lakes, NJ). To analyze cell apoptosis, the drug pre-treated cells were washed with ice-cold PBS twice and harvested with trypsin, then resuspended with 5 μl Annexin V-FITC/PI (BD, San Jose, CA) mixture. After incubated at room temperature for 20 min in the dark condition, the cells were measured by BD Accuri TM C6 flow cytometer (BD, Franklin Lakes, NJ). To detect the total intracellular ROS generation, cells were collected and washed with pre-warmed PBS, then stained with 10 μM DCFH-DA in DMEM at 37 °C for 30 min in the dark condition. Cells were collected and flow cytometer was used to measure the DCFH-DA fluorescence.

### Colony formation assay

MDA-MB-231 and Hs578T cells were seeded in 6-well plates at low density 4 × 10^3^ cells/well and cultured with curcumin (0.1 μg/mL) or WZ35 (0.5 μg/mL) for 14 days till visible colonies appeared. The cells were then stained with crystal violet. Colony number was calculated by Image J software. The test was repeated 3 times.

### Transwell migration and invasion assays

In vitro cell migration and invasion assays were performed using a 24-well transwell (Coring, USA) with an 8-μm pore polycarbonate membrane as previously described [21]. Briefly, for the invasion assay, the top portion of the chambers were precoated with Matrigel (BD Biosciences) diluted with FBS free media (1: 20). The chambers without Matrigel were used for migration assay. Twenty thousand cells were added into the top chamber with 200 μL serum-free medium, after incubation at 37 °C for 2 h, curcumin (10 μg/mL) or WZ35 (10 μg/mL) was added to the top compartment of the chambers. Then 500 μL complete medium were filled in the lower chambers. After being incubated at 37 °C and allowed to migrate for 24 h, non-migratory or non-invasive cells above the upper chambers were removed with cotton swabs. The cells migrated or invade stuck to the lower transwell surfaces were fixed with 4% paraformaldehyde and stained with crystal violet for 3 min. The cells were imaged and counted in five fields of vision observed using a microscope with 20x magnification.

### Measurement of oxidative phosphorylation

The Seahorse XF96 Extracellular Flux Analyser (Seahorse Bioscience, North Billerica, MA, USA) was used to detect real time integrated cellular oxygen consumption rate (OCR) according to the manufacturer’s protocol. In brief, MDA-MB-231 cells were treated with or without drugs for 12 h and 1 × 10^3^ cells were plated into the seahorse customized cell plates. After baseline measurements, the OCR was detected with sequential injection of oligomycin (ATP synthase inhibitor; 1 μM), FCCP (uncoupler; 0.5 μM), rotenone (complex I inhibitor; 1 μM), and antimycin A (complex III inhibitor; 1 μM).

### Quantitative RT-PCR

Total RNA was extracted with Trizol reagent (Invitrogen) from MDA-MB-231 cells pre-treated with curcumin and WZ35 according to the manufacturer’s instructions. RNA integrity was confirmed using spectrophotometry and formaldehyde/agarose gel electrophoresis. 1000 ng RNA was reverse transcribed into cDNA using the Prime Script TM RT reagent Kit with gDNA Eraser (Takara, Dalian, China). Quantitative real-time PCR assays were performed on a CFX connect TM real-time system (Bio-Rad) using SYBR Green (Bio-Rad) according to the manufacturer’s protocol. Each sample was replicated for three times. All results are expressed as means ±SD.

### Western blot analysis

Cells and tissue samples were washed with PBS and lysed in RIPA lysis Buffer (Beyotime, Jiangsu, China) supplemented with protease inhibitors (Complete, EDTA-free; Roche, USA) and then centrifuged at 12000 rpm for 10 min. Equal amounts of protein lysates (50 μg each) were separated by 10% SDS-PAGE and transferred to PVDF membranes (EMD Millipore, Burlington, MA, USA). The Membranes were blocked with 5% non-fat milk in PBS with 0.05% Tween 20 (PBST) at room temperature for 1.5 h and incubated overnight with primary antibodies (1: 1000) at 4 °C. After wash with PBST for 5 min three times, the membranes were incubated with corresponding secondary antibodies (1:2000) for 1 h at room temperature. After extensive washing, the protein bands were detected using ECL kit (Bio-Rad, Hercules, CA).

### Immunohistochemistry

Tissue sections were initially deparaffinized with xylene, rehydrated, and antigen retrieval was performed in 0.01 M citrate buffer (PH = 6.0) for 3 min at 95 °C. Followed by incubation with primary antibody at 4 °C overnight, the tissue sections were incubated with secondary antibody at room temperature for 2 h. Finally, after DAB staining and a neutral gum sealing, immunohistochemical signals were photographed and observed under a microscope (Olympus Corporation, Tokyo, Japan) with magnification of 200 × .

### Animal experiments

Five-week-old, athymic BALB/c nu/nu female mice (16-19 g, totally *n* = 24) were purchased from Vital River Laboratories (Beijing, China). The mice were randomly divided into three groups. MDA-MB-231 cells were subcutaneously inoculated into the right flank of mice (1 × 10^7^ cells in 100 ul PBS per mouse). After tumor volume reached 50 mm^3^, the mice were intraperitoneally injected Castor oil, curcumin and WZ35 (0.2 mL, 25 mg/kg for each) for 15 days. The tumor size and body weight of nude mice were measured and recorded once every other day. The tumor volumes were determined by measuring length (L) and width (W) and calculating volume (V = 0.5 × L × W^2^) at the indicated time points. At the end of experiment, the animals were sacrificed and the tumors were harvested for use in proteins expression and histology studies. To investigate the effects of curcumin or WZ35 on lung metastasis in vivo, 4 × 10^5^ of MDA-MB-231 cells in 100 ul of PBS were intravenously injected into the mice. After 24 days, 24 nude mice were randomly divided into four groups (normal saline, castor oil, curcumin, WZ35) and treated mice for 21 days. The lung metastases were evaluated by microscope after HE staining.

### Statistical analysis

All experiments were conducted in triplicate (*n* = 3) and the data are presented as the mean ± SD. All statistical analyses were processed with GraphPad Prism 6.0 (GraphPad Software, La Jolla, CA, USA). Two-sided student’s t-test was performed to analyze the differences between two groups of data. *P* value< 0.05 means statistically significant.

## Results

### WZ35 exhibits stronger anti-tumor activities than curcumin in the breast cancer cells

**Fig. 1:**
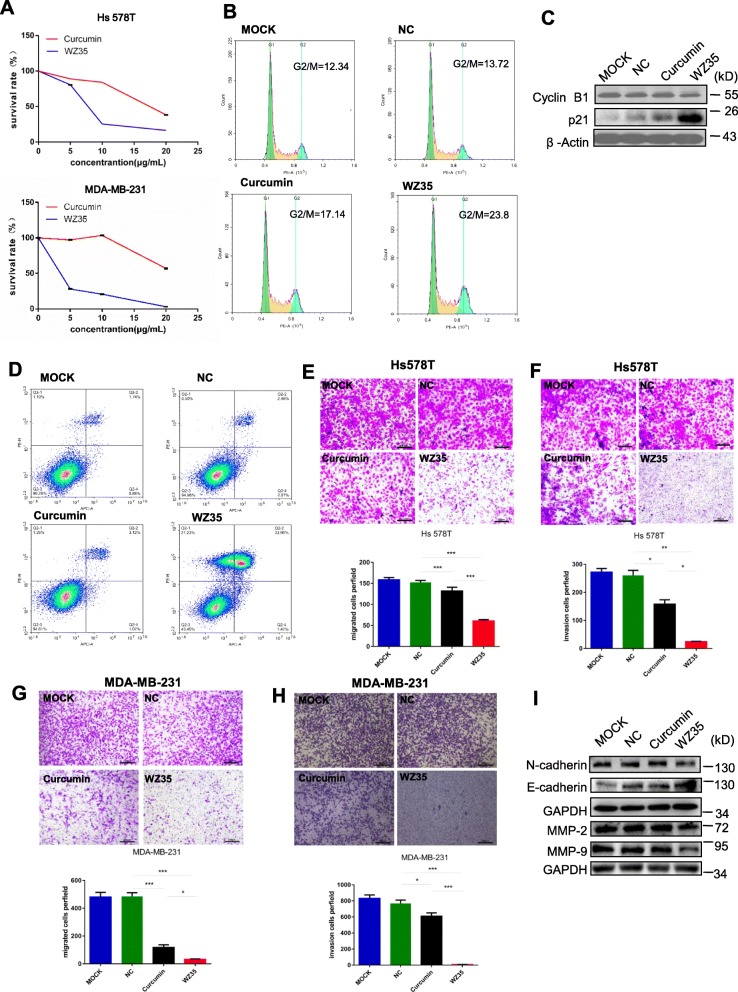
WZ35 inhibits breast cancer cells proliferation, migration, invasion and induced apoptotic cell death and cell cycle arrest. **a** The effects of WZ35 and curcumin on the proliferation of breast cancer cells. **b** Induction of cycle arrest in MDA-MB-231 cells was detected by Flow cytometry after treatment with curcumin (10 μg/mL) and WZ35 (10 μg/mL) for 24 h. WZ53 increased the proportion of cells in the G2/M phase. **c** Expression of cell cycle relative proteins Cyclin B1, and p21 were determined by western blot after treatment with WZ35 (10 μg/mL) or curcumin (10 μg/mL) for 24 h. GAPDH was used as internal control. **d** Induction of apoptosis in MDA-MB-231 cells was determined by flow cytometry after treatment with WZ35 (10 μg/mL) and curcumin (10 μg/mL) for 24 h. Similar results were obtained in three independent experiments. **e**, **h** Transwell assay was performed to evaluate the effects of WZ35 and curcumin on Hs578T (**e**, **f**) and MDA-MB-231 (**g**, **h**) cells migration and invasion. Cell migration and invasion images were presented and migrated cells were quantified. **i** EMT biomakers N-cadherin, E-cadherin, MMP-2 and MMP-9 were determined by western blot. Data are presented as mean ± SD, *p < 0.05, **p < 0.01, ***p < 0.001

We firstly evaluated the effects of WZ35 on the proliferation of breast cancer cell lines, Hs578T and MDA-MB-231 by using CCK8 assay. After treatment of cells with WZ35 or curcumin for 24 h, we found that WZ35 significantly reduced cell survival rate in a dose-dependent manner with stronger anti-proliferative effects than curcumin (Fig. 1a). Next, we tested the effects of WZ35 on the cell proliferation of human mammary gland epithelial cell, MCF10A and human normal lung epithelial cell, BEAS-2B. WZ35 effectively inhibited MDA-MB-231 cell proliferation at low concentration with IC50 around 1 μg/ml. In contrast, WZ35 showed less effects to both MCF10A and BEAS-2B cells compare with MDA-MB-231 cells with IC50 > 7.5 μg/ml (Additional file 1: Figure S1A and B). We further analyzed the effects of WZ35 on the cell cycle. As shown in Fig. 1b and Additional file 1: Figure S2A, treatment of MDA-MB-231 cells with WZ35 significantly increased the proportion of cells in the G2/M phase compared with control group and curcumin. Accordingly, cell cycle G2/M phase related proteins including Cyclin B1 and p21 were markedly changed (Fig. 1c). In addition, we evaluated the effect of WZ35 on the apoptosis in breast cancer cells by Annexin V-FITC/PI-staining. Our results showed that treatment of cells with WZ35 (10 μg/mL) significantly increased the ratio of apoptotic cells compared with that curcumin (10 μg/mL) and control group (Fig. 1d and Additional file 1: Figure S2B). Furthermore, colony formation assay showed that treatment of cells with WZ35 (0.5 μg/mL) markedly reduced the number of colonies of MDA-MB-231 and Hs578T cells (Additional file 1: Figure S2C-D). Since the metastasis is the major cause of high mortality in breast cancer, we conducted transwell assay after treatment of Hs578T and MDA-MB-231 cells with WZ35 to evaluate the effects of WZ35 on the cell migration and invasion. Treatment of cells with curcumin or WZ35 significantly reduced migratory and invasive ability of Hs578T (Fig. 1e, f) and MDA-MB-231 (Fig. 1g, h and Additional file 1: Figure S2E-F) cells. As expected, WZ35 showed stronger anti-migratory and anti-invasive abilities than curcumin. Consistent with these findings, we found that the expression of epithelial-mesenchymal transition (EMT) related proteins were also significantly changed. In detail, the expressions of mesenchymal markers (N-cadherin, MMP-2 and MMP-9) were reduced; whereas the expression of epithelial marker, E-cadherin was increased (Fig. 1i). All together, these data indicate that WZ35 plays stronger tumor suppressive role than curcumin in breast cancer cells.

### WZ35 suppresses MDA-MB-231 xenograft tumor growth and metastasis in vivo

**Fig. 2:**
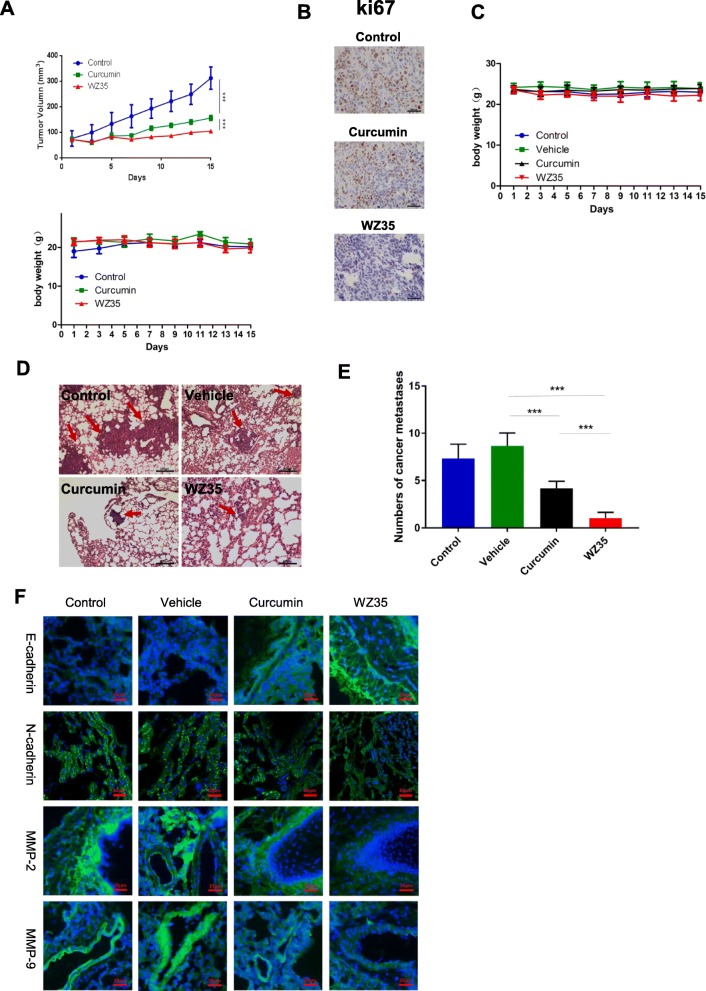
WZ35 inhibits MDA-MB-231 xenograft tumor growth and metastasis in vivo. **a** A growth curve analysis of the tumor growth in curcumin, WZ35 or Castor oil treated groups. **b** Expression of ki67 in tumor tissues was determined by immunohistochemistry. **c** Mice body weight was measured during the 17-day treatment of WZ35 and curcumin. **d** Representative microscopic images of lung metastatic lesions at 21 days after treatment of mice with curcumin or WZ35 (0.2 mL, 25 mg/kg for each). **e** The number of lung metastatic tumors was calculated. **f** Immunofluorescence were used to test the expression level of E-cadherin, N-cadherin, MMP-2 and MMP-9 from curcumin, WZ35, Vehicle and control group. Data are presented as mean ± SD, ***p < 0.001

Based on the aforementioned results from in vitro experiments, we questioned whether WZ35 also has anti-tumor effects in vivo. To confirm our hypothesis, we developed a xenograft mouse model by injecting MDA-MB-231 cells into the right frank of 5 weeks old female nude mice. Similar with the in vitro experiments, treatment of mice with curcumin or WZ35 (0.2 mL, 25 mg/kg for each) showed significantly reduced tumor volume compared to the control mice. In agreement with in vitro analysis, WZ35 showed stronger anti-tumor activities than curcumin in vivo with no major change of body weight suggesting that the WZ35 would not lead to significant cytotoxicity (Fig. 2a). Immunohistochemistry demonstrated that WZ35 treatment markedly reduced the expression of Ki-67 in tumor tissues indicating that WZ35 inhibited cell proliferation in vivo (Fig. 2b). To further determine the anti-metastatic activities of WZ35 in vivo, we intravenously injected MDA-MB-231 cells into the nude mice and evaluated lung metastasis foci by HE analysis. As shown in Fig. 2c-e, both curcumin and WZ35 significantly reduced the numbers of lung metastasis foci, and WZ35 showed more superior anti-metastatic effects than curcumin without major change of body weight. Immunofluorescence analyses validated the expression of mesenchymal markers, N-cadherin, MMP-2 and MMP-9 were suppressed in mice tumor tissues; whereas the expression of epithelial marker, E-cadherin was increased in mice tumor tissues after treatment of mice with WZ35 (Fig. 2f). As expected, WZ35 showed more strong effects than curcumin. These data further confirmed that the anti-metastatic activities of WZ35 both in vitro and in vivo.

### YAP and JNK pathways are involved in WZ35 mediated breast cancer cell growth inhibition

**Fig. 3:**
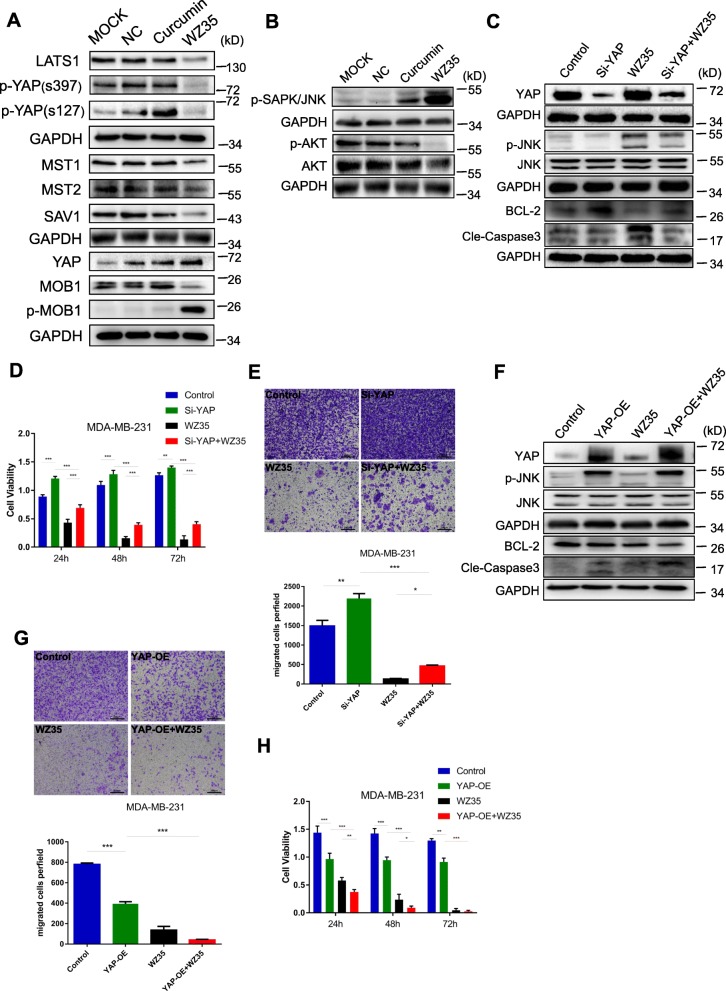
YAP and JNK pathway are involved in WZ35 mediated breast cancer cell growth inhibition. **a** Western blot analysis of the Hippo pathway related proteins MST1, MST2, LATS1, MOB1, p-MOB1, p-YAP(s397), p-YAP(s127), YAP/TAZ and SAV1 in MDA-MB-231 cells treated with curcumin (10 μg/mL) or WZ35 (10 μg/mL). GAPDH was used as loading control. **b** Western blot analysis of p-JNK, AKT and p-AKT expression in MDA-MB-231 cells treated with WZ35 (10 μg/mL) or curcumin (10 μg/mL). **c** MDA-MB-231 cells were transfected with or without YAP siRNA and co-treated with WZ35 (10 μg/mL) for 12 h, the protein expression of YAP, JNK, p-JNK, BCL-2 and cleaved caspase-3 were determined by western blot. **d** MDA-MB-231 cells were transfected with or without YAP siRNA and co-treated with WZ35 (10 μg/mL) for 24 h, 48 h, 72 h respectively. Then cell viability was detected by CCK8 assay. **e** Representative images and the numbers of migrated cells were evaluated after treatment of MDA-MB-231 cells with Si-YAP, WZ35 and Si-YAP+WZ35. **f** Western blot analysis of YAP, JNK, p-JNK, BCL-2 and Cle-Caspase3 protein levels in MDA-MB-231 cells treated with YAP-OE, WZ35 and YAP-OE + WZ35. **g** Representative images and the numbers of migrated cells were evaluated after treatment of MDA-MB-231 cells with YAP-OE, WZ35 and YAP-OE + WZ35. **h** MDA-MB-231 cells were transfected with or without YAP-OE and co-treated with WZ35 (10 μg/mL) for 24 h, 48 h, 72 h respectively. The cell viability was detected by CCK8 assay. Data are presented as mean ± SD, *p < 0.05, **p < 0.01, ***p < 0.001

We next set out to determine the potential mechanism of the anti-tumor activities of WZ35 in breast cancer cells. Yap has been reported to interact with DNA-binding protein TEAD transcription factors to promote the expression of growth-promoting and apoptosis-inhibiting genes [22]. The Ser/Thr kinase Hippo (Mst1 and Mst2) activates Lats1/2, which phosphorylate YAP and sequester YAP in cytoplasm to inhibit transcriptional activity of YAP [23–25]. We found that treatment of MDA-MB-231 cells with WZ35 significantly reduced the expression of MST1/2, Lats1, p-YAP(s397), p-YAP(s127) as well as hippo pathway scaffolding proteins SAV1 and MOB1; whereas increased the expression of YAP with stronger effects than curcumin (Fig. 3a). JNK has been reported to play an essential role in Hippo signaling induced cell invasion [26]. In addition, it is widely known that JNKs are involved in cell proliferation, apoptosis, inflammation, differentiation and migration [27, 28]. The JNK activation could inhibit anti-apoptotic proteins, BCL-2 and BCL-xl expression; whereas induces pro-apoptotic protein BAX expression [29–31]. To further examine the association of YAP signaling and JNK in the WZ35 mediated breast cancer cells growth inhibition, we performed a series of knockdown and overexpression analyses. As shown in Fig. 3b, treatment of MDA-MB-231 cells with WZ35 markedly increased phospho-JNK expression, whereas decreased phospho-AKT expression. Importantly, knockdown of YAP by siRNA attenuated WZ35 induced p-JNK expression. YAP knockdown also elevated anti-apoptotic protein BCL-2 expression, whereas reduced cleaved caspase-3 expression. Furthermore, treatment of YAP knockdown cells with WZ35 significantly attenuated the effects of WZ35 on the expression of BCL-2 and cleaved caspase-3 (Fig. 3c). Accordingly, knockdown of YAP in MDA-MB-231 cells promoted cell proliferation and migration, and co-treatment of cells with YAP siRNA and WZ35 partly reversed tumor suppressive effects of WZ35 (Fig. 3d, e). As expected, we observed that the opposite effects in YAP overexpressing cells for p-JNK, BCL-2 and cleaved-CASP3 expression (Fig. 3f). Treatment of YAP overexpressing cells with WZ35 exhibited stronger anti-proliferative and anti-migratory effects than YAP overexpression or WZ35 treatment alone (Fig. 3g, h). Taken together, our data suggest that WZ35 inhibits breast cancer cell growth and migration through constitutive YAP activation and subsequent JNK phosphorylation.

### WZ35 induced ROS generation is responsible for YAP and JNK activation

**Fig. 4:**
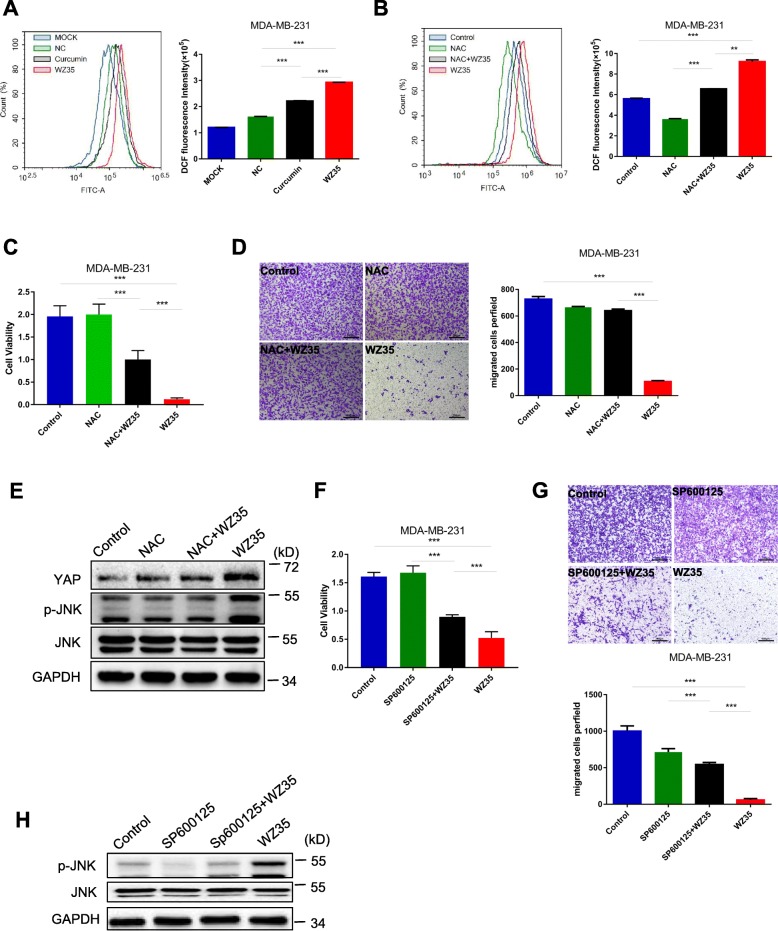
WZ35 induces cytotoxicity in breast cancer cells is depending on ROS mediated YAP and JNK activation. **a** Flow cytometry analysis were performed to determine the intracellular ROS levels in MDA-MB-231 cells treated with curcumin or WZ35. **b** Intracellular ROS levels in MDA-MB-231 cells treated with WZ35 or WZ35 + NAC. **c** Cell proliferation ability was detected by CCK8 assay. **d** Representative images and the numbers of migration cells were evaluated after treatment of MDA-MB-231 cells with NAC, WZ35 and NAC + WZ35. **e** The expression of YAP, JNK and p-JNK in MDA-MB-231 cells treated with NAC, WZ35 and NAC + WZ35 were detected by western blot. **f** Cell proliferation ability was detected by CCK8 assay. **g** Representative images and the numbers of migrated cells were evaluated after treatment of MDA-MB-231 cells with SP600125, WZ35 and SP600125 + WZ35. **h** The expression of JNK and p-JNK proteins were determined by western blot. Data are presented as mean ± SD, **p < 0.01, ***p < 0.001

Zou et al. has demonstrated that ROS generation is the upstream regulator of WZ35-induced apoptosis in gastric cancer [13]. Therefore, we attempt to determine whether the anti-tumor effects of WZ35 in MDA-MB-231 cells were associated with its oxidative stress. As shown in Fig. 4a, treatment of MDA-MB-231 cells with WZ35 increased ROS generation in a dose dependent manner. In addition, pretreatment of MDA-MB-231 cells with N-acetyl cysteine (ROS scavenger; NAC, 1 mM) significantly reduced WZ35-induced increases in DCF fluorescence as expected (Fig. 4b). Next, we wanted to check whether the negative growth and migratory signals from WZ35 can be attenuated by reduction of ROS levels. Pretreatment of cells with NAC (1 mM) was able to attenuate WZ35 induced anti-proliferative (Fig. 4c) and migratory (Fig. 4d) ability in MDA-MB-231 cells. Moreover, NAC prevented WZ35 induced alternations of YAP and p-JNK expression (Fig. 4e) indicating that WZ35-induced ROS generation is responsible for YAP and JNK activation mediated anti-tumor activities in breast cancer cells. The JNK activation plays critical role in various cancer cell apoptosis. It has been reported that JNK induces apoptosis via phosphorylating 14–3-3 protein, which in turn releases pro-apoptotic proteins, such as BAX and FOXO transcription factors [32–35]. To further demonstrate the importance of JNK activation in WZ35 mediated anti-tumor activities, MDA-MB-231 cells were pre-treated with a JNK inhibitor, SP600125. Our results showed that SP600125 significantly attenuated anti-proliferative (Fig. 4f) and migratory effects (Fig. 4g) of WZ35 in MDA-MB-231 cells. Accordingly, we validated that SP600125 inhibited WZ35 induced p-JNK expression (Fig. 4h). These results indicate that the WZ35-induced anti-tumor activities in MDA-MB-231 cells at least partially mediated by ROS-YAP-JNK signaling pathway.

### ROS-YAP-JNK pathway is involved in WZ35 induced mitochondrial dysfunction

**Fig. 5:**
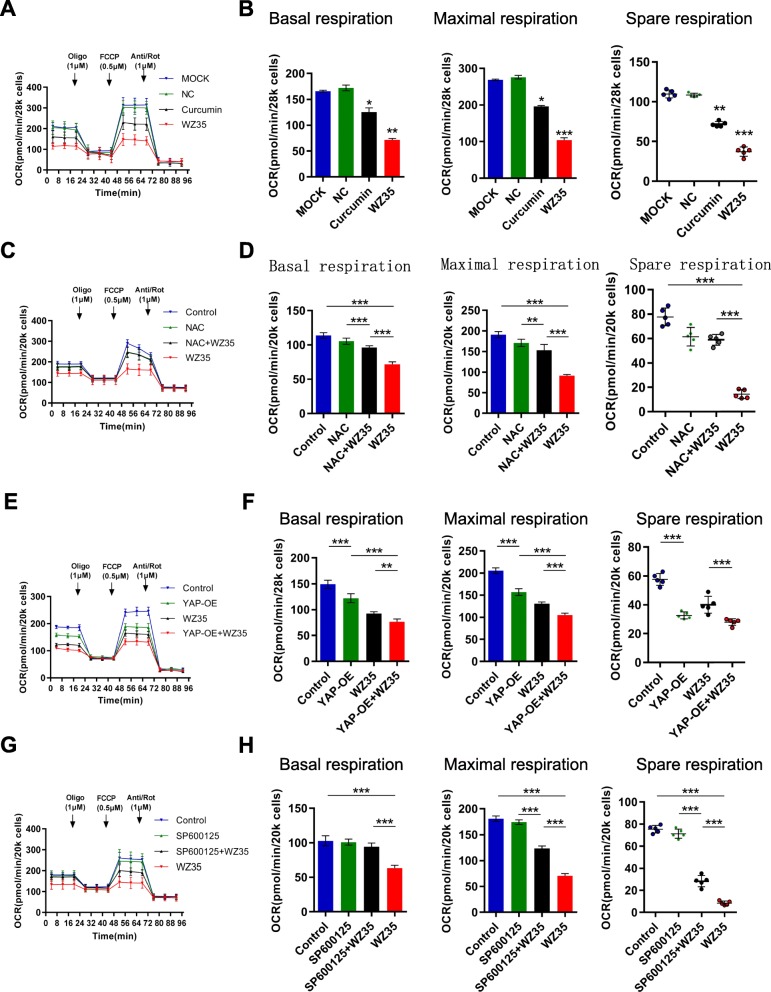
ROS-YAP-JNK pathway is involved in WZ35 induced mitochondrial dysfunction. **a** MDA-MB-231 cells were cultured with WZ35 or curcumin for 12 h, and cellular oxygen consumption rate (OCR) was measured in real time using the Seahorse XF96 Extracellular Flux Analyzer after basal OCR was measured at three time points, followed by sequential injection of oligomycin (1 μM), FCCP (0.5 μM) rotenone (1 μM), and antimycin A (1 μM). The overall OCR curves were plotted as the mean OCR ± SD of three replicates. **b** Basal respiration, maximal respiration and spare respiration were assessed, respectively. **c**, **d** MDA-MB-231 cells were treated with or without WZ35 and co-treated with NAC. **e**, **f** MDA-MB-231 cells were overexpressed YAP and treated with or without WZ35. **g**, **h** MDA-MB-231 cells were treated with or without WZ35 and co-treated with JNK inhibitor SP600125. The OCR was measured in real time using the Seahorse XF96 Extracellular Flux Analyzer as aforementioned. The Basal respiration, maximal respiration and spare respiration were also assessed, respectively. Data are presented as the mean ± SD, *p < 0.05, **p < 0.01, ***p < 0.001

Mitochondrial dysfunction is a common phenomenon in multiple human malignancies, such as breast cancer [36], HCC [37], lung cancer [38]. Moreover, mitochondrial fission is important for ROS production [39]. Firstly, to determine the effects of WZ35 in mitochondrial dysfunction, we evaluated mitochondrial DNA transcription, replication and translation associated proteins including nuclear respiratory factor 1 (NRF1) and NRF2, DNA polymerase subunit gamma (POLG) and mitochondrial translation factor EF4 after treatment of cells with WZ35. As shown in Additional file 1: Figure S3A-B, WZ35 significantly down-regulates the expression of mitochondria-associated proteins EF-4, PLOG, NRF1 and NRF2 in MDA-MB-231 cells. Moreover, WZ35 markedly reduced NRF1 and NRF2 mRNA levels in MDA-MB-231 cells (Additional file 1: Figure S3C-D). Secondly, to further investigate the effects of WZ35 on mitochondrial respiration, we evaluated real-time oxidative phosphorylation by measuring cellular oxygen consumption rates (OCRs). WZ35 significantly decreased OCR compared with curcumin and negative control (NC) group in MDA-MB-231 cells suggesting that WZ35 treatment led to inhibit mitochondrial respiration (Fig. 5a). We further analyzed major parameters of mitochondria function by evaluating OCR data at various time points. WZ35 markedly decreased basal respiration, maximal respiration, spare respiration (Fig. 5b) and ATP production in MDA-MB-231 cells (Additional file 1: Figure S4A). Importantly, co-treatment of MDA-MB-231 cells with ROS inhibitor, NAC and WZ35 significantly attenuated inhibitory effects of WZ35 on OCR as well as basal respiration, maximal respiration and spare respiration suggesting that WZ35-induced inhibition of mitochondrial respiration was mediated by ROS generation (Fig. 5c, d). To further investigate whether ROS mediated YAP and JNK activation were involved in the WZ35 induced mitochondrial dysfunction, we performed a series of experiments. First, we overexpressing or knockdown YAP in MDA-MB-231 cells and treated cells with WZ35. YAP overexpression significantly reduced OCR, basal respiration, maximal respiration and spare respiration. Moreover, Treatment of YAP overexpressing MDA-MB-231 cells with WZ35 exerts stronger effects than either YAP overexpression or WZ35 treatment alone (Fig. 5e, f). Inversely, knockdown of YAP increased OCR, basal respiration, maximal respiration and spare respiration, and treatment of YAP knockdown cells with WZ35 significantly attenuated inhibitory effects of WZ35 on basal respiration, maximal respiration and spare respiration in MDA-MB-231 cells (Additional file 1: Figure S4B-C). Second, we treated MDA-MB-231 cells with JNK inhibitor (SP600125) and WZ35. As expected, co-treatment of cells with SP600125 and WZ35 significantly attenuated inhibitory effects of WZ35 on OCR, basal respiration, maximal respiration and spare respiration (Fig. 5g, h). Taken all together, these data indicate that ROS-YAP-JNK pathway is involved in WZ35 induced mitochondrial dysfunction.

### YAP expression is down-regulated in breast cancer and correlated with the prognosis of certain breast cancer patients

**Fig. 6:**
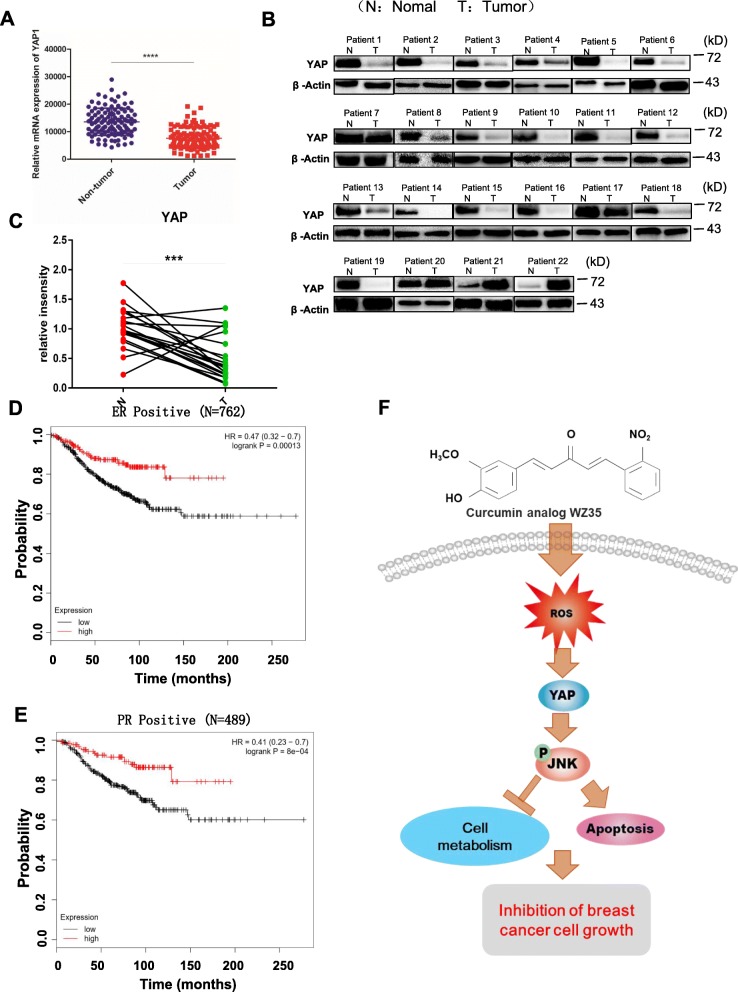
YAP plays a tumor suppressive functions in breast cancer. **a** YAP1 mRNA expression in breast cancer tissues and normal adjacent tissues from TCGA RNA-seq data. **b** The protein levels of YAP in twenty two breast cancer tissues and normal tissues were measured by western blot. **c** Comparison of YAP1 expression in 22 paired breast cancer tissues and normal adjacent tissues by qRT-PCR. **d** and **e** Kaplan-Meier plots of overall survival of ER positive breast cancer patients (*N* = 762) (**d**) and PR positive breast cancer patients (*N* = 489) (**e**) expressing high and low levels of YAP. Data obtained from the Kaplan-Meier plotter database (kmplot.com/analysis). **f** Schematic illustration for the underlying anti-cancer mechanism of WZ35 in breast cancer. Data are presented as the mean ± SD, ***p < 0.001, ****p < 0.0001

Treatment of breast cancer cells with WZ35 exhibited strong anti-tumor activities through activating YAP mediated JNK signaling suggesting that potential tumor suppressive role of YAP in breast cancer. To further investigate the functions of YAP in breast cancer tissue specimens, we evaluated YAP mRNA levels in breast cancer tissues using TCGA public database. As shown in Fig. 6a, analysis of 226 paired breast cancer tissues and normal adjacent tissues (NATs) for which YAP mRNA expression was available in the TCGA dataset, demonstrated YAP mRNA was significantly down-regulated in breast cancer tissues compare to the NATs. We further examined YAP protein levels in 22 paired fresh breast cancer tissues samples and corresponding NATs by western blot analysis. Among 22 paired patient samples, 19 patient samples showed significantly reduced YAP protein expression levels in breast cancer tissue samples compared with corresponding NATs (Fig. 6b). We also checked mRNA levels of YAP in these 22 pairs of primary breast cancer tissues and their adjacent tissue counterparts by qRT-PCR. As presented in Fig. 6c, YAP mRNA was significantly down regulated in primary breast cancer tissue specimens compared with their adjacent noncancerous tissues. To further investigate whether downregulated YAP expression in breast cancer is associated with patient’s survival, we performed Kaplan-Meier survival analysis by breast cancer subtypes. We found that high expression of YAP was significantly associated with favorable prognosis of ER positive breast cancer patients (*N* = 762) (Fig. 6d) and PR positive breast cancer patients (*N* = 489) (Fig. 6e). Inversely, high expression of YAP was significantly associated with poor prognosis of triple negative breast cancers (*N* = 161) (Additional file 1: Figure S5). Altogether, these results imply that YAP plays tumor suppressive functions in breast cancer and associated with the prognosis of certain breast cancer patients by their ER, PR and Her2 status. Further studies need to validate this result using large number of breast cancer patient samples.

## Discussion

Currently, the mainstay of systemic treatment for breast cancer is endocrine therapy. However, chemotherapy still is indispensable especially for patients they are lacking hormone receptors [3]. Thus, discover novel drugs and therapeutic targets become a matter of great interest. WZ35, an analog of curcumin, has been demonstrated to exhibit superior anti-tumor effects in gastric cancer and hepatocellular carcinoma [13, 14]. However, anti-tumor effects of WZ35 in breast cancer and its underlying anti-tumor mechanisms are still unclear. Here, we demonstrated that WZ35 exhibits superior anti-tumor effects compare to the curcumin on breast cancer cells both in vitro and in vivo with no evident side effects. Further analysis showed that ROS mediated YAP and JNK activation were involved in WZ35 induced anti-tumor activities. In addition, we found that ROS-YAP-JNK pathway is implicated in mitochondria dysfunction in breast cancer cells (Fig. 6f).

Of note, curcumin has been reported to stimulate immune systems to exert anti-cancer activity [10, 40]. In our animal study, curcumin showed less in vitro anti-tumor activities, however, it is quite effective in vivo mice xenograft model suggesting possible immune stimulatory effect of curcumin. Although we used immune compromised mice to compare anti-tumor efficacy of WZ35 and curcumin, considering that NK cells and macrophages do still exist in these mice, WZ35 may have had some immune stimulatory activity which might contribute to its anti-tumor activities in vivo although further study needs to validate this proposal.

The Hippo pathway is associated with cell proliferation, tissue homeostasis and tumorigenesis [19, 41]. The components of Hippo pathway (Mst1/2, Lats1/2 etc.) have been reported to play tumor suppressive roles in cancers. YAP is a key downstream effector of Hippo pathway, which has been reported to act as either an oncogene or tumor suppressor in breast cancer [42]. Moreover, YAP was reported to act as a tumor-suppressor in lung SCC via disruption of intracellular ROS homeostasis [43]. In our study, we demonstrated that YAP exerts tumor suppressive role in breast cancer cells. WZ35 induced YAP activation was involved in inhibition of breast cancer cell growth and migration. We also found that YAP mRNA and protein levels were markedly down-regulated in breast cancer tissue specimens and reduced YAP expression was associated with poor prognosis of certain type of breast cancer patients. Accumulated ROS generation in cancer has been reported to inhibit cell proliferation [44], induce DNA damage [45], autophagy [46, 47], cellular injury [44], cell death [43, 46] and drug resistance [48, 49]. In addition, previous research demonstrated that elevated ROS production could activate JNK signaling pathway, resulting in cell apoptosis [50, 51]. Cancer cells with increased oxidative stress are likely to be more vulnerable to the damage. Therefore, elevated ROS production in cancer cells that do not cause significant toxicity to normal cells might be a potential therapeutic method. Our study showed that WZ35 induced ROS generation in breast cancer cells and NAC effectively attenuated the anti-tumor activities of WZ35. We also found for the first time that WZ35 mediated ROS generation is involved in YAP and JNK activation in breast cancer cells. Several studies have highlighted that activation of JNK signaling pathway induces mitochondria-dependent cell apoptosis via activating downstream signaling molecules of BCL-2 family proteins and caspase-3 [52, 53]. We demonstrated that YAP activation exerts anti-tumor activities via JNK phosphorylation and activation, and subsequent BCL-2 downregulation and cle-caspase3 upregulation.

Mitochondria plays critical role in the cells through regulation of energy metabolism, ATP generation, and calcium homeostasis [54, 55]. Accumulated evidences have indicated that mitochondrial metabolism is an attractive target for cancer therapy. Emerging studies have begun to demonstrate that mitochondria takes part in the activation of signaling pathways including the PI3K pathway, and activation of oncogenes such as MYC and KRAS, which result in promoting cell proliferation [56]. The mitochondrial ATP is mainly generated by glycolysis and mitochondrial oxidative phosphorylation. It has been reported that both glycolytic and mitochondrial functions were decreased by either KRAS suppression or ERK inhibition [57]. In this study, we further investigated relationship between ROS-YAP-JNK pathway activation and mitochondrial dysfunction, and found that WZ35 inhibits mitochondrial oxidative phosphorylation by activating ROS-YAP-JNK pathway. Activation of ROS-YAP-JNK pathway not only induced apoptosis, but also accelerated mitochondrial dysfunction by possibly inhibiting ATP generation in breast cancer cells resulting in inhibition of tumor growth. Thus, activating ROS-YAP-JNK pathway might increase the sensitivity of breast cancer cells to anticancer drug. Further study needs to prove this matter.

Altogether, our study shows that WZ35 exhibits stronger anti-tumor activities than curcumin by activating ROS-YAP-JNK signaling pathway. The activated ROS-YAP-JNK pathway involved in anti-tumor activities of breast cancer cells by inducing mitochondrial dysfunction and apoptosis. These results suggest that novel therapeutic strategies for breast cancer by targeting ROS-YAP-JNK pathway.

## Conclusion

Our study shows that WZ35 exhibits stronger anti-tumor activities than curcumin by activating ROS-YAP-JNK signaling pathway. Our study revealed novel ROS-YAP-JNK pathway which involved in anti-tumor activities of breast cancer cells by inducing mitochondrial dysfunction and apoptosis. These results suggest that novel therapeutic strategies for breast cancer by targeting ROS-YAP-JNK pathway.

## Supplementary information

**Additional file 1: Figure S1.** The effects of WZ35 on cell proliferation of normal cells. **Figure S2.** The effects of WZ35 on cell cycle arrest, apoptosis, migration and invasion ability. **Figure S3.** W35 significantly down-regulates the expression of mitochondria-associated proteins. **Figure S4.** WZ35 induces mitochondrial dysfunction. **Figure S5.** Kaplan-Meier plot of overall survival of triple negative breast cancer patients expressing high and low levels of YAP.

## Data Availability

All data generated or analyzed in this study are included in this manuscript and its additional files.

## Abbreviations

ATP: Adenosine triphosphate
BCL-2: B cell lymphoma 2
DMEM: Dulbecco’s modified eagle medium
EF4: mitochondrial translation factor
EMT: Epithelial-mesenchymal transition
ER: Estrogen receptor
ERK: Extracellular regulated protein kinases
FBS: Fetal bovine serum
FCCP: Carbonylcyanide-p-trifluorometh oxyphenylhydrazone
HCC: Hepatocellular carcinoma
HRP: Horseradish peroxidase
JNK: c-Jun N-terminal kinase
KRAS: Kirsten rat sarcoma viral onco
LATS1: Large tumour suppressor 1
MMP-2: Matrix Metalloproteinase-2
MMP-9: Matrix Metalloproteinase-9
MST1: Mammalian STE20-like protein kinase 1
MST2: Mammalian STE20-like protein kinase 2
NAC: N -Acetyl-L-cysteine
NATs: Normal adjacent tissues
NC: Negative control
NRF1: Nuclear respiratory factor 1
NRF2: Nuclear respiratory factor 2
OCR: Oxygen consumption rate
OD: Optical density
PBS: Phosphate buffer solution
PI: Propidium Iodide
POLG: DNA polymerase subunit gamma
PR: Progesterone receptor
ROS: Reactive oxygen species
SAV1: Salvador family WW domain-containing protein 1
TCGA: The Cancer Genome Atlas
YAP: Yes-associated protein
