# Correction: Curcumin derivative WZ35 inhibits tumor cell growth via ROS-YAP-JNK signaling

**DOI:** 10.1186/s13046-026-03813-4

**Published:** 2026-08-29

**Authors:** Lihua Wang, Canwei Wang, Zheying Tao, Liqian Zhao, Zheng Zhu, Wencan Wu, Ye He, Hong Chen, Bin Zheng, Xiangjie Huang, Yun Yu, Linjun Yang, Guang Liang, Ri Cui, Tongke Chen

**Affiliations:** 1 School of Ophthalmology and Optometry, Eye Hospital, Wenzhou Medical University, Wenzhou, 325000 Zhejiang China; 2 State Key Laboratory of Ophthalmology, Optometry and Visual Science, Wenzhou, 325000 Zhejiang China; 3 Affiliated Yueqing Hospital and School of Pharmaceutical Sciences, Wenzhou Medical University, Wenzhou, 325035 Zhejiang China; 4 School of Medicine, Shanghai Jiaotong University, Shanghai, 200030 China; 5 Laboratory Animal Centre, Wenzhou Medical University, Wenzhou, Zhejiang China; 6 School of Pharmaceutical Sciences, Wenzhou Medical University, Wenzhou, 325035 Zhejiang China

**Correction: J Exp Clin Cancer Res 38, 460 (2019)**

**https://doi.org/10.1186/s13046-019-1424-4**

Following publication of the original article [1], the authors identified inappropriate reuse of GAPDH loading control bands in Figs. 1I and 3A, and 3B. The affected figure panels have been replaced with the correct versions.

Incorrect Fig. 1I

**Fig. 1:**
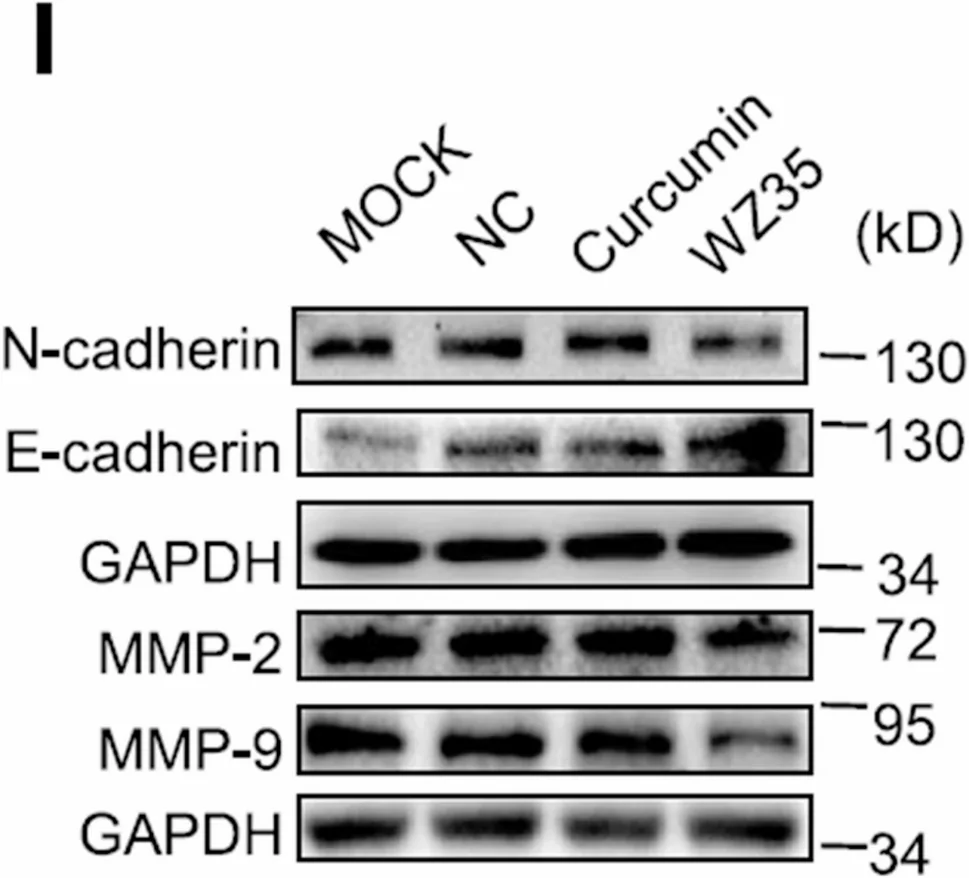
WZ35 inhibits breast cancer cells proliferation, migration, invasion and induced apoptotic cell death and cell cycle arrest. **a** The effects of WZ35 and curcumin on the proliferation of breast cancer cells. **b** Induction of cycle arrest in MDA-MB-231 cells was detected by Flow cytometry after treatment with curcumin (10 µg/mL) and WZ35 (10 µg/mL) for 24 h. WZ53 increased the proportion of cells in the G2/M phase. **c** Expression of cell cycle relative proteins Cyclin B1, and p21 were determined by western blot after treatment with WZ35 (10 µg/mL) or curcumin (10 µg/mL) for 24 h. GAPDH was used as internal control. **d** Induction of apoptosis in MDA-MB-231 cells was determined by flow cytometry after treatment with WZ35 (10 µg/mL) and curcumin (10 µg/mL) for 24 h. Similar results were obtained in three independent experiments. **e**, **h** Transwell assay was performed to evaluate the effects of WZ35 and curcumin on Hs578T (**e**, **f**) and MDA-MB-231 (**g**, **h**) cells migration and invasion. Cell migration and invasion images were presented and migrated cells were quantified. **i** EMT biomakers N-cadherin, E-cadherin, MMP-2 and MMP-9 were determined by western blot. Data are presented as mean ± SD, *p < 0.05, **p < 0.01, ***p < 0.001

Correct Fig. 1I

**Fig. 1:**
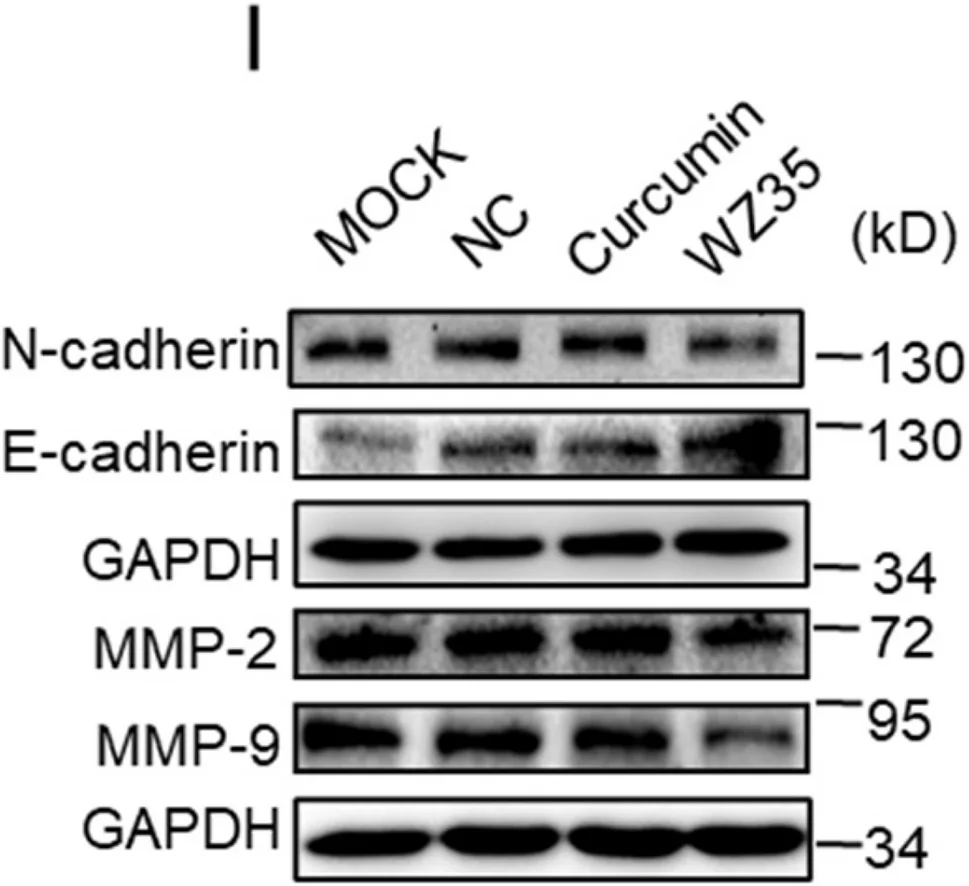
WZ35 inhibits breast cancer cells proliferation, migration, invasion and induced apoptotic cell death and cell cycle arrest. **a** The effects of WZ35 and curcumin on the proliferation of breast cancer cells. **b** Induction of cycle arrest in MDA-MB-231 cells was detected by Flow cytometry after treatment with curcumin (10 µg/mL) and WZ35 (10 µg/mL) for 24 h. WZ53 increased the proportion of cells in the G2/M phase. **c** Expression of cell cycle relative proteins Cyclin B1, and p21 were determined by western blot after treatment with WZ35 (10 µg/mL) or curcumin (10 µg/mL) for 24 h. GAPDH was used as internal control. **d** Induction of apoptosis in MDA-MB-231 cells was determined by flow cytometry after treatment with WZ35 (10 µg/mL) and curcumin (10 µg/mL) for 24 h. Similar results were obtained in three independent experiments. **e**, **h** Transwell assay was performed to evaluate the effects of WZ35 and curcumin on Hs578T (**e**, **f**) and MDA-MB-231 (**g**, **h**) cells migration and invasion. Cell migration and invasion images were presented and migrated cells were quantified. **i** EMT biomakers N-cadherin, E-cadherin, MMP-2 and MMP-9 were determined by western blot. Data are presented as mean ± SD, *p < 0.05, **p < 0.01, ***p < 0.001

Incorrect Fig. 3A

**Fig. 3:**
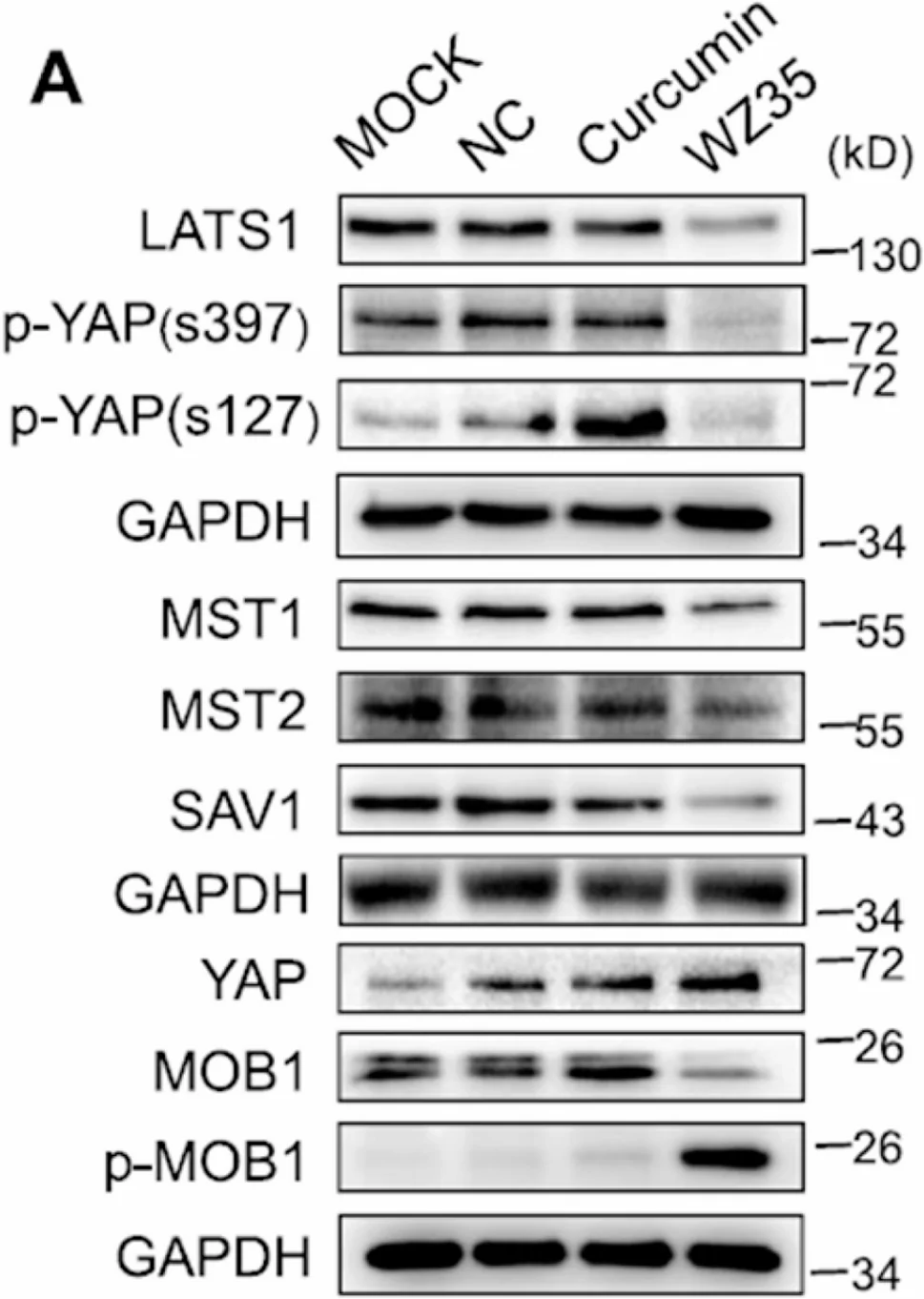
YAP and JNK pathway are involved in WZ35 mediated breast cancer cell growth inhibition. **a** Western blot analysis of the Hippo pathway related proteins MST1, MST2, LATS1, MOB1, p-MOB1, p-YAP(s397), p-YAP(s127), YAP/TAZ and SAV1 in MDA-MB-231 cells treated with curcumin (10 µg/mL) or WZ35 (10 µg/mL). GAPDH was used as loading control. **b** Western blot analysis of p-JNK, AKT and p-AKT expression in MDA-MB-231 cells treated with WZ35 (10 µg/mL) or curcumin (10 µg/mL). **c** MDA-MB-231 cells were transfected with or without YAP siRNA and co-treated with WZ35 (10 µg/mL) for 12 h, the protein expression of YAP, JNK, p-JNK, BCL-2 and cleaved caspase-3 were determined by western blot. **d** MDA-MB-231 cells were transfected with or without YAP siRNA and co-treated with WZ35 (10 µg/mL) for 24 h, 48 h, 72 h respectively. Then cell viability was detected by CCK8 assay. **e** Representative images and the numbers of migrated cells were evaluated after treatment of MDA-MB-231 cells with Si-YAP, WZ35 and Si-YAP+WZ35. **f** Western blot analysis of YAP, JNK, p-JNK, BCL-2 and Cle-Caspase3 protein levels in MDA-MB-231 cells treated with YAP-OE, WZ35 and YAP-OE + WZ35. **g** Representative images and the numbers of migrated cells were evaluated after treatment of MDA-MB-231 cells with YAP-OE, WZ35 and YAP-OE + WZ35. **h** MDA-MB-231 cells were transfected with or without YAP-OE and co-treated with WZ35 (10 µg/mL) for 24 h, 48 h, 72 h respectively. The cell viability was detected by CCK8 assay. Data are presented as mean ± SD, *p < 0.05, **p < 0.01, ***p < 0.001

Correct Fig. 3A

**Fig. 3:**
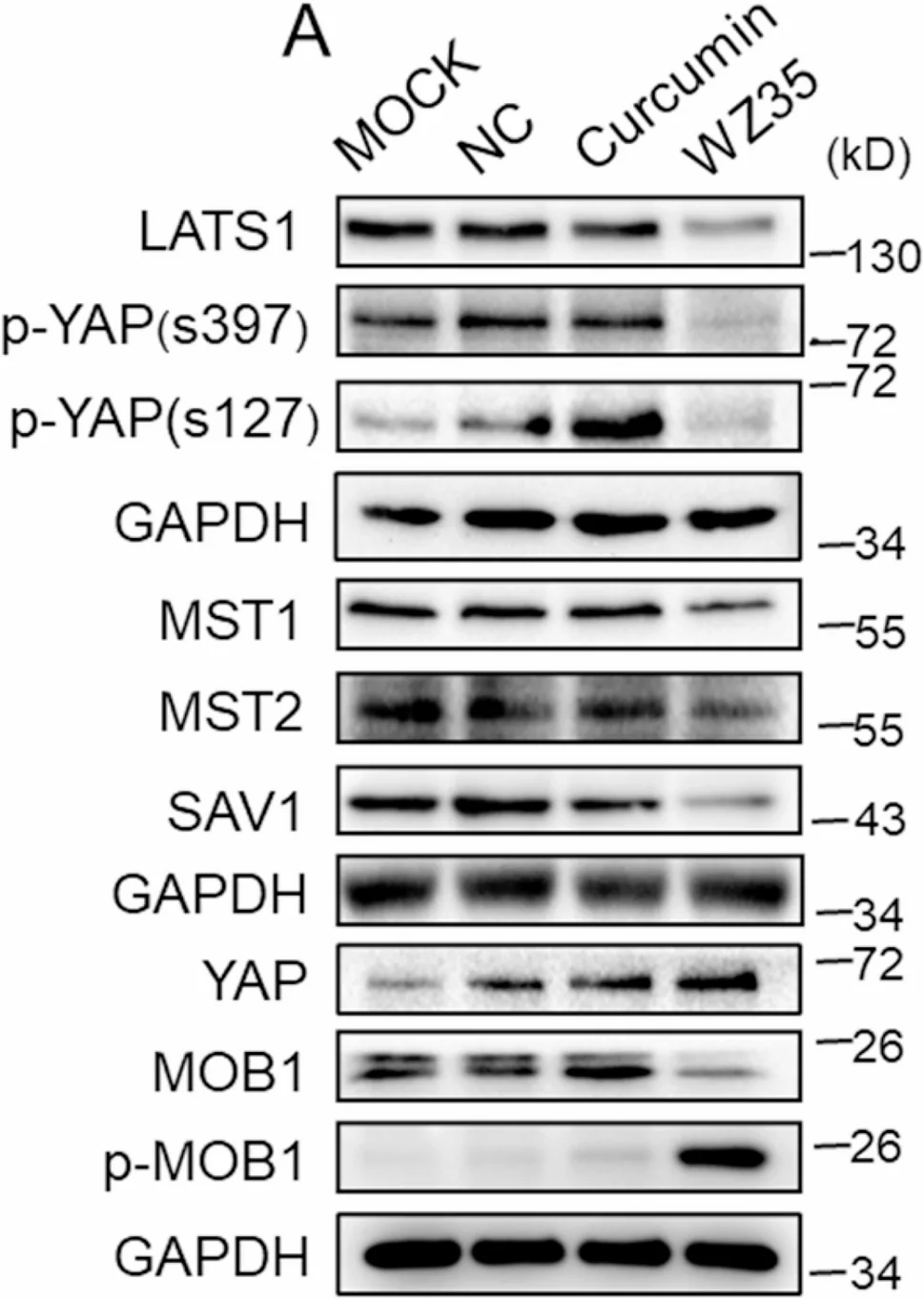
YAP and JNK pathway are involved in WZ35 mediated breast cancer cell growth inhibition. **a** Western blot analysis of the Hippo pathway related proteins MST1, MST2, LATS1, MOB1, p-MOB1, p-YAP(s397), p-YAP(s127), YAP/TAZ and SAV1 in MDA-MB-231 cells treated with curcumin (10 µg/mL) or WZ35 (10 µg/mL). GAPDH was used as loading control. **b** Western blot analysis of p-JNK, AKT and p-AKT expression in MDA-MB-231 cells treated with WZ35 (10 µg/mL) or curcumin (10 µg/mL). **c** MDA-MB-231 cells were transfected with or without YAP siRNA and co-treated with WZ35 (10 µg/mL) for 12 h, the protein expression of YAP, JNK, p-JNK, BCL-2 and cleaved caspase-3 were determined by western blot. **d** MDA-MB-231 cells were transfected with or without YAP siRNA and co-treated with WZ35 (10 µg/mL) for 24 h, 48 h, 72 h respectively. Then cell viability was detected by CCK8 assay. **e** Representative images and the numbers of migrated cells were evaluated after treatment of MDA-MB-231 cells with Si-YAP, WZ35 and Si-YAP+WZ35. **f** Western blot analysis of YAP, JNK, p-JNK, BCL-2 and Cle-Caspase3 protein levels in MDA-MB-231 cells treated with YAP-OE, WZ35 and YAP-OE + WZ35. **g** Representative images and the numbers of migrated cells were evaluated after treatment of MDA-MB-231 cells with YAP-OE, WZ35 and YAP-OE + WZ35. **h** MDA-MB-231 cells were transfected with or without YAP-OE and co-treated with WZ35 (10 µg/mL) for 24 h, 48 h, 72 h respectively. The cell viability was detected by CCK8 assay. Data are presented as mean ± SD, *p < 0.05, **p < 0.01, ***p < 0.001

Incorrect Fig. 3B

**Fig. 3:**
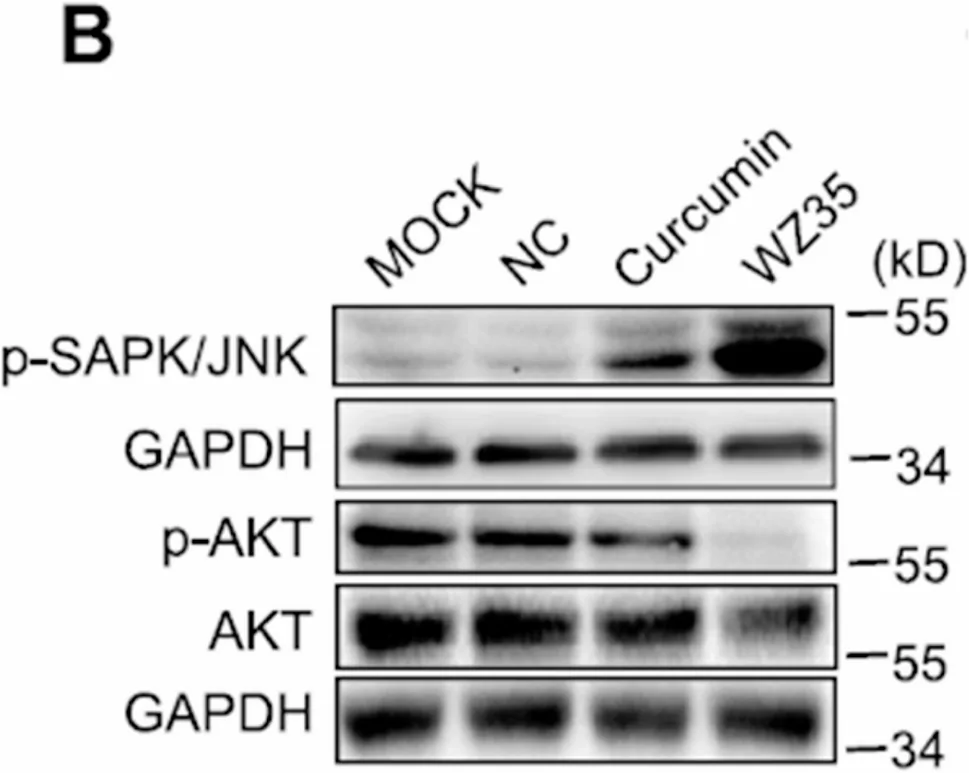
YAP and JNK pathway are involved in WZ35 mediated breast cancer cell growth inhibition. **a** Western blot analysis of the Hippo pathway related proteins MST1, MST2, LATS1, MOB1, p-MOB1, p-YAP(s397), p-YAP(s127), YAP/TAZ and SAV1 in MDA-MB-231 cells treated with curcumin (10 µg/mL) or WZ35 (10 µg/mL). GAPDH was used as loading control. **b** Western blot analysis of p-JNK, AKT and p-AKT expression in MDA-MB-231 cells treated with WZ35 (10 µg/mL) or curcumin (10 µg/mL). **c** MDA-MB-231 cells were transfected with or without YAP siRNA and co-treated with WZ35 (10 µg/mL) for 12 h, the protein expression of YAP, JNK, p-JNK, BCL-2 and cleaved caspase-3 were determined by western blot. **d** MDA-MB-231 cells were transfected with or without YAP siRNA and co-treated with WZ35 (10 µg/mL) for 24 h, 48 h, 72 h respectively. Then cell viability was detected by CCK8 assay. **e** Representative images and the numbers of migrated cells were evaluated after treatment of MDA-MB-231 cells with Si-YAP, WZ35 and Si-YAP+WZ35. **f** Western blot analysis of YAP, JNK, p-JNK, BCL-2 and Cle-Caspase3 protein levels in MDA-MB-231 cells treated with YAP-OE, WZ35 and YAP-OE + WZ35. **g** Representative images and the numbers of migrated cells were evaluated after treatment of MDA-MB-231 cells with YAP-OE, WZ35 and YAP-OE + WZ35. **h** MDA-MB-231 cells were transfected with or without YAP-OE and co-treated with WZ35 (10 µg/mL) for 24 h, 48 h, 72 h respectively. The cell viability was detected by CCK8 assay. Data are presented as mean ± SD, *p < 0.05, **p < 0.01, ***p < 0.001

Correct Fig. 3B

**Fig. 3:**
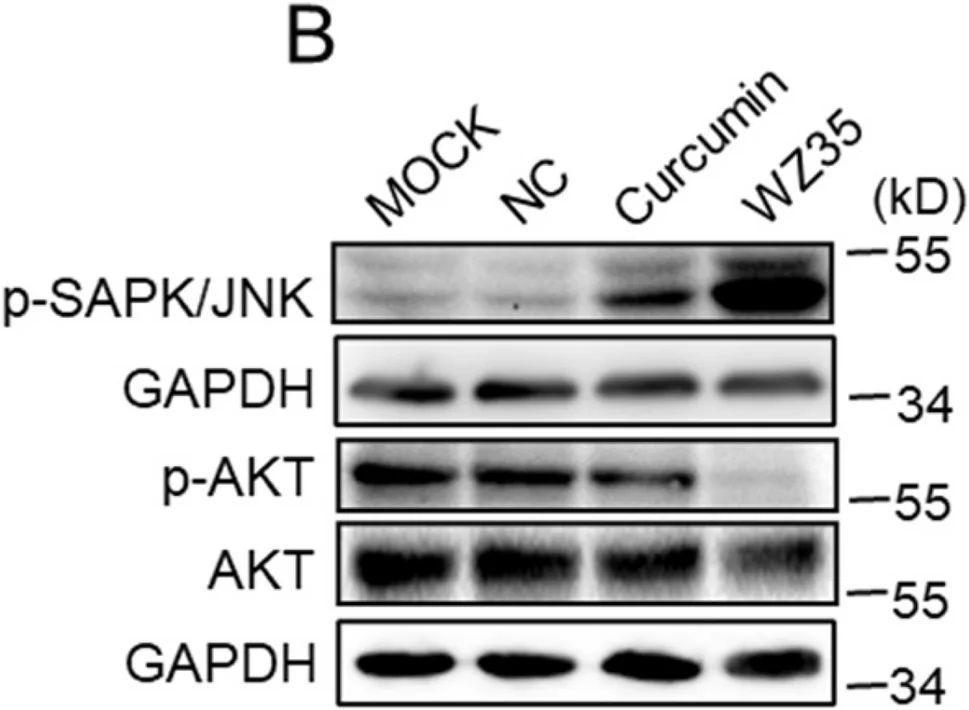
YAP and JNK pathway are involved in WZ35 mediated breast cancer cell growth inhibition. **a** Western blot analysis of the Hippo pathway related proteins MST1, MST2, LATS1, MOB1, p-MOB1, p-YAP(s397), p-YAP(s127), YAP/TAZ and SAV1 in MDA-MB-231 cells treated with curcumin (10 µg/mL) or WZ35 (10 µg/mL). GAPDH was used as loading control. **b** Western blot analysis of p-JNK, AKT and p-AKT expression in MDA-MB-231 cells treated with WZ35 (10 µg/mL) or curcumin (10 µg/mL). **c** MDA-MB-231 cells were transfected with or without YAP siRNA and co-treated with WZ35 (10 µg/mL) for 12 h, the protein expression of YAP, JNK, p-JNK, BCL-2 and cleaved caspase-3 were determined by western blot. **d** MDA-MB-231 cells were transfected with or without YAP siRNA and co-treated with WZ35 (10 µg/mL) for 24 h, 48 h, 72 h respectively. Then cell viability was detected by CCK8 assay. **e** Representative images and the numbers of migrated cells were evaluated after treatment of MDA-MB-231 cells with Si-YAP, WZ35 and Si-YAP+WZ35. **f** Western blot analysis of YAP, JNK, p-JNK, BCL-2 and Cle-Caspase3 protein levels in MDA-MB-231 cells treated with YAP-OE, WZ35 and YAP-OE + WZ35. **g** Representative images and the numbers of migrated cells were evaluated after treatment of MDA-MB-231 cells with YAP-OE, WZ35 and YAP-OE + WZ35. **h** MDA-MB-231 cells were transfected with or without YAP-OE and co-treated with WZ35 (10 µg/mL) for 24 h, 48 h, 72 h respectively. The cell viability was detected by CCK8 assay. Data are presented as mean ± SD, *p < 0.05, **p < 0.01, ***p < 0.001
