# Supplementary material for: Curcumin derivative WZ35 inhibits tumor cell growth via ROS-YAP-JNK signaling pathway in breast cancer
Source: J Exp Clin Cancer Res. 2019 Nov 8;38:460. doi: 10.1186/s13046-019-1424-4 (PMC6842168; doi:10.1186/s13046-019-1424-4)
Supplement: Supplementary file 1 — Additional file 1: Figure S1. The effects of WZ35 on cell proliferation of normal cells. Figure S2. The effects of WZ35 on cell cycle arrest, apoptosis, migration and invasion ability. Figure S3. W35 significantly down-regulates the expression of mitochondria-associated proteins. Figure S4. WZ35 induces mitochondrial dysfunction. Figure S5. Kaplan-Meier plot of overall survival of triple negative breast cancer patients expressing high and low levels of YAP. [file 13046_2019_1424_MOESM1_ESM.pdf]

Supplementary figures

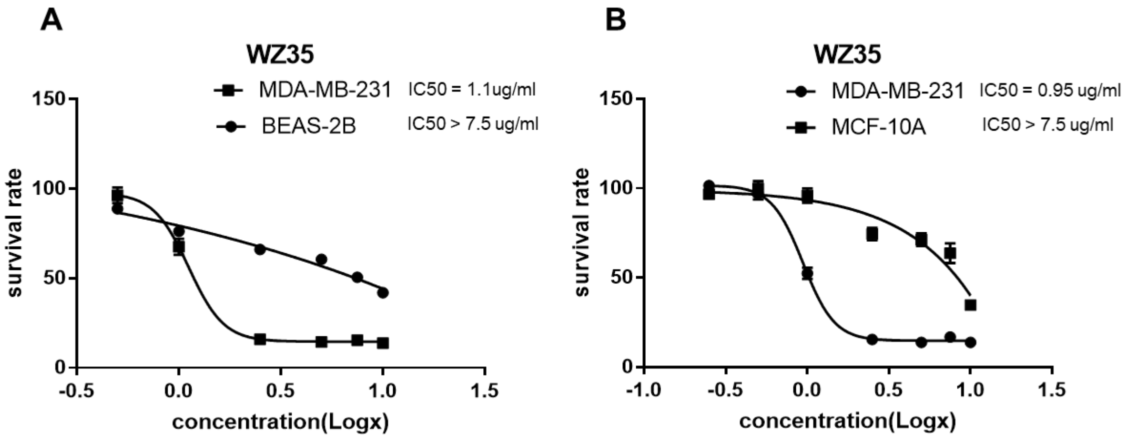

Figure S1. The effects of WZ35 on cell proliferation of normal cells. Cytotoxicity of WZ35 towards BEAS-2B cells (A) and MCF-10A cells (B) was assessed using the MTT assay to compare with cytotoxicity of WZ35 towards MDA-MB-231 cells. The assays were independently reproduced three times, with n = 3 in each independent experiment.

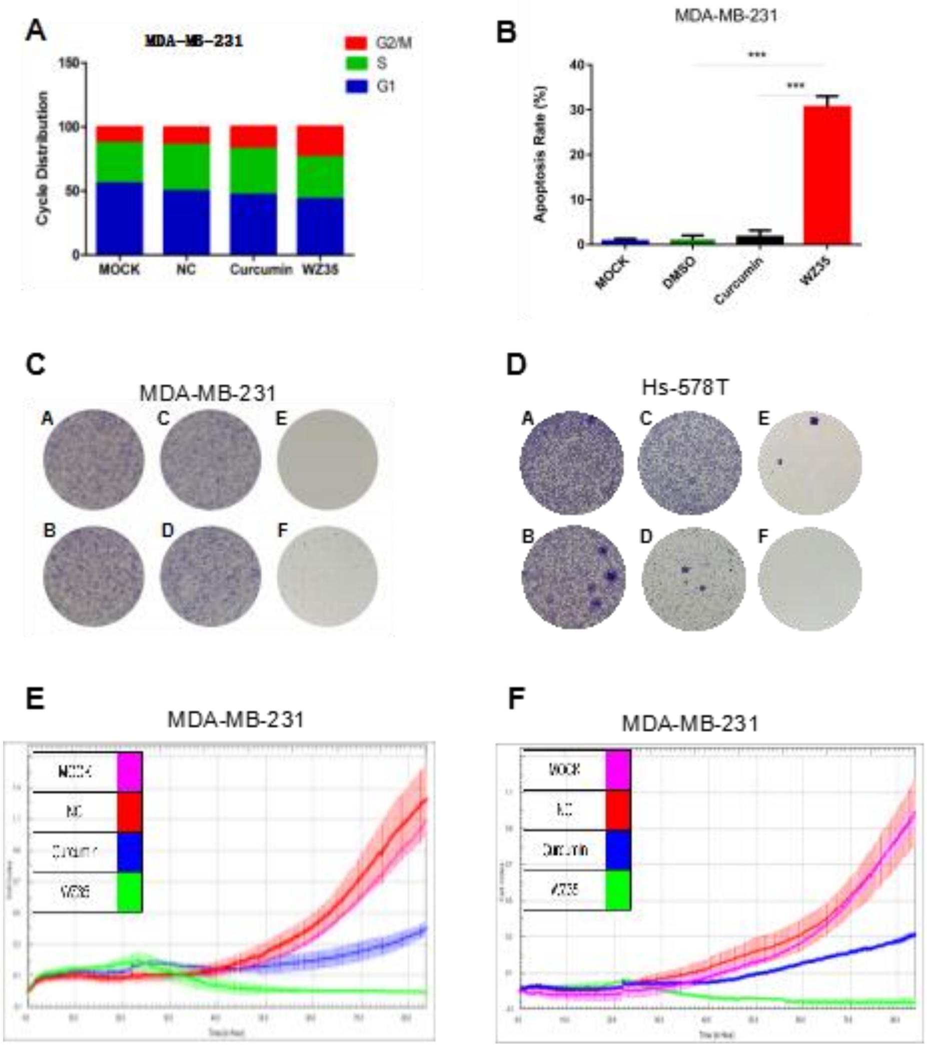

Figure S2. The effects of WZ35 on cell cycle arrest, apoptosis, migration and invasion ability. (A) WZ35 increased the proportion of cell at the G2/M phase. The ratio of each cell cycle phase was assayed by GraphPad Prism. (B) The percentage of apoptotic cells in each group was calculated. (C-D) Colony formation analyses of MDA-MB-231 (C) and Hs-578T cells (D) after the treatment of cells with A: MOCK, B:NC, C: Curcumin 0.1 μg/mL, D: Curcumin 0.5μg/mL, E: WZ35 0.1 μg/mL, F: WZ35 0.5μg/mL. (E-F) The overall cell migration and invasion abilities of MDA-MB-231 cells was detected by RTCA.

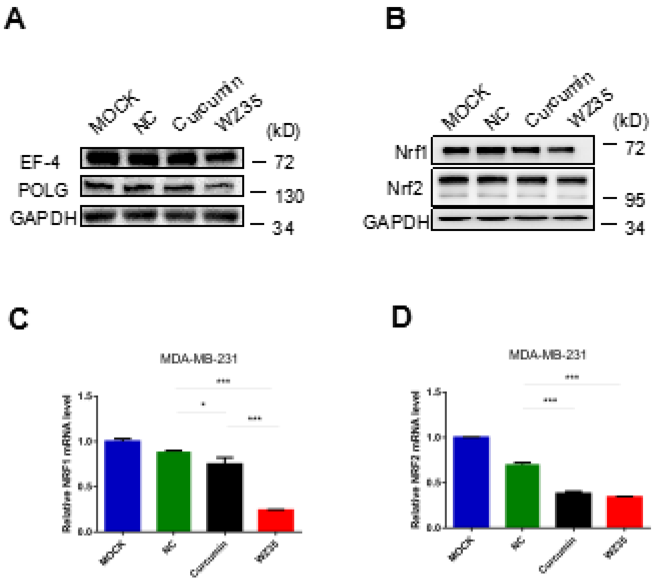

Figure S3. W35 significantly down-regulates the expression of mitochondria-associated proteins. (A) Western blot analysis of the protein levels of EF-4 and POLG in MDA-MB-231 cells after treatment with Curcumin or WZ35. (B-D) The protein levels (B) and mRNA levels (C and D) of NRF1 and NRF2 were determined by western blot and RT-PCR, respectively. *P < 0.05, ***P < 0.001.

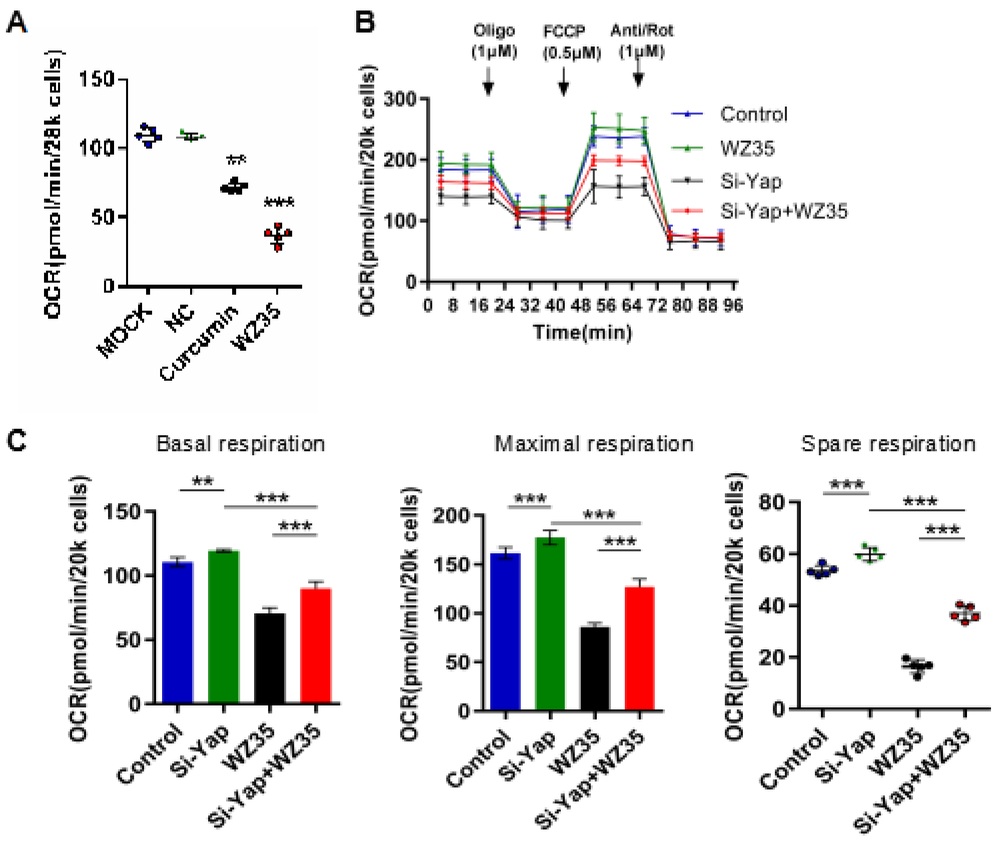

Figure S4. WZ35 induces mitochondrial dysfunction. (A) ATP production was measured after treatment of MDA-MB-231cells with curcumin or WZ35. (B-C) MDA-MB-231 cells were treated with siYAP, WZ35 and siYAP+WZ35. OCR was measured in real time using the Seahorse XF96 Extracellular Flux Analyzer after basal OCR was measured at three time points, followed by sequential injection of oligomycin (1 μM), FCCP (0.5 μM) rotenone (1 μM), and antimycin A (1 μM). The overall OCR curves were plotted as the mean OCR ± SD of three replicates (B). Basal respiration, maximal respiration and spare respiration were also measured (C). Data are presented as the mean ± SD, ***P* < 0.01, ****P* < 0.001.

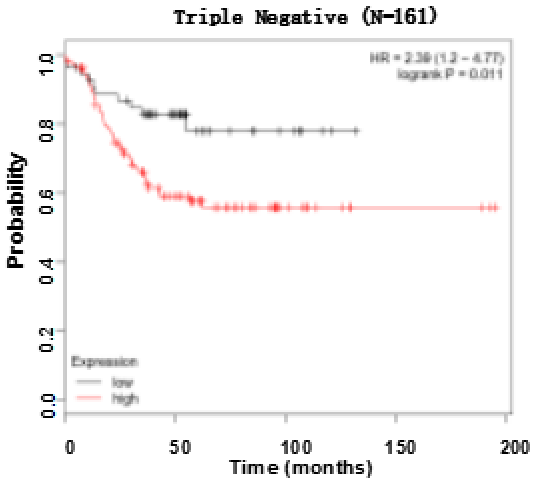

Figure S5. Kaplan-Meier plot of overall survival of triple negative breast cancer patients (N=161) expressing high and low levels of YAP. Data obtained from the Kaplan-Meier plotter database (kmplot.com/analysis).
